# Ectopic splenic torsion—a challenging diagnosis in a resource-limited setting mimicking a gynecologic emergency: a case report

**DOI:** 10.1093/jscr/rjaf182

**Published:** 2025-04-04

**Authors:** Ashenafi A Feleke, Muluken A Zemariam, Suleiman A Belay, Ayalkebet T Worku, Getachew S Yigezaw, Nigat A Addis, Eyasu F Yitina

**Affiliations:** Department of Surgery, School of Medicine, College of Medicine and Health Sciences, University of Gondar, Maraki Street, Gondar City, Central Gondar Zone, PO Box 196, Gondar, Ethiopia; Department of Surgery, School of Medicine, College of Medicine and Health Sciences, University of Gondar, Maraki Street, Gondar City, Central Gondar Zone, PO Box 196, Gondar, Ethiopia; School of Medicine, College of Medicine and Health Sciences, University of Gondar, Maraki Street, Gondar City, Central Gondar Zone, PO Box 196, Gondar, Ethiopia; Department of Surgery, School of Medicine, College of Medicine and Health Sciences, University of Gondar, Maraki Street, Gondar City, Central Gondar Zone, PO Box 196, Gondar, Ethiopia; Department of Gynecology and Obstetrics, School of Medicine, College of Medicine and Health Sciences, University of Gondar, Maraki Street, Gondar City, Central Gondar Zone, PO Box 196, Gondar, Ethiopia; Department of Gynecology and Obstetrics, School of Medicine, College of Medicine and Health Sciences, University of Gondar, Maraki Street, Gondar City, Central Gondar Zone, PO Box 196, Gondar, Ethiopia; Department of Gynecology and Obstetrics, School of Medicine, College of Medicine and Health Sciences, University of Gondar, Maraki Street, Gondar City, Central Gondar Zone, PO Box 196, Gondar, Ethiopia

**Keywords:** splenectomy, splenic infarction, splenopexy, wandering spleen

## Abstract

A 35-year-old woman presented with severe lower abdominal pain, a history of intermittent abdominal discomfort, increased abdominal girth, and weight loss over the past year. Physical examination revealed a tender abdomen with guarding and a palpable lower abdominal mass. Initial ultrasound suggested a torsed adnexal mass, prompting emergency laparotomy, which revealed a torsed, infarcted wandering spleen. Splenectomy was performed, and the patient recovered well with appropriate post-splenectomy care. Wandering spleen is a rare condition characterized by hypermobility and ectopic positioning of the spleen, often asymptomatic but potentially presenting emergently with torsion and infarction. Diagnosis is challenging, particularly in resource-limited settings, but imaging such as ultrasound or computed tomography (CT) scans can aid in identification. Surgical intervention, including splenopexy or splenectomy, is essential in acute cases.

## Introduction

Wandering spleen is a rare condition where the spleen becomes hypermobile and ectopic due to congenital or acquired causes. While often asymptomatic, it can present with chronic pain or, as in this case, as an acute emergency due to torsion. Surgical intervention, including splenopexy or splenectomy, is required in emergent cases. Diagnosis is typically confirmed through imaging such as ultrasound, CT scans, or magnetic resonance imaging (MRI). This case report describes a 35-year-old woman initially presenting as a gynecologic emergency, later diagnosed with splenic torsion following surgical exploration.

## Case presentation

A 35-year-old multiparous woman from Northern Gondar, Ethiopia, presented with severe lower abdominal pain lasting 2 weeks. She reported intermittent mild abdominal pain, increased abdominal girth, loss of appetite, and weight loss over the past year. Her menstrual cycle was regular, and she denied other gynecologic complaints.

On examination, her vital signs were stable: blood pressure 110/70 mmHg, pulse rate 108 beats/min, temperature 36.9°C, and respiratory rate 22 breaths/min. The abdomen was diffusely tender with guarding, and a palpable lower abdominal mass was noted. Laboratory tests, including complete blood count and organ function tests, were normal. Abdominopelvic ultrasound revealed a hypoechoic intraperitoneal mass (13.3 × 5.75 mm) in the pelvic area, with high suspicion of left adnexal origin.

**Figure 1:**
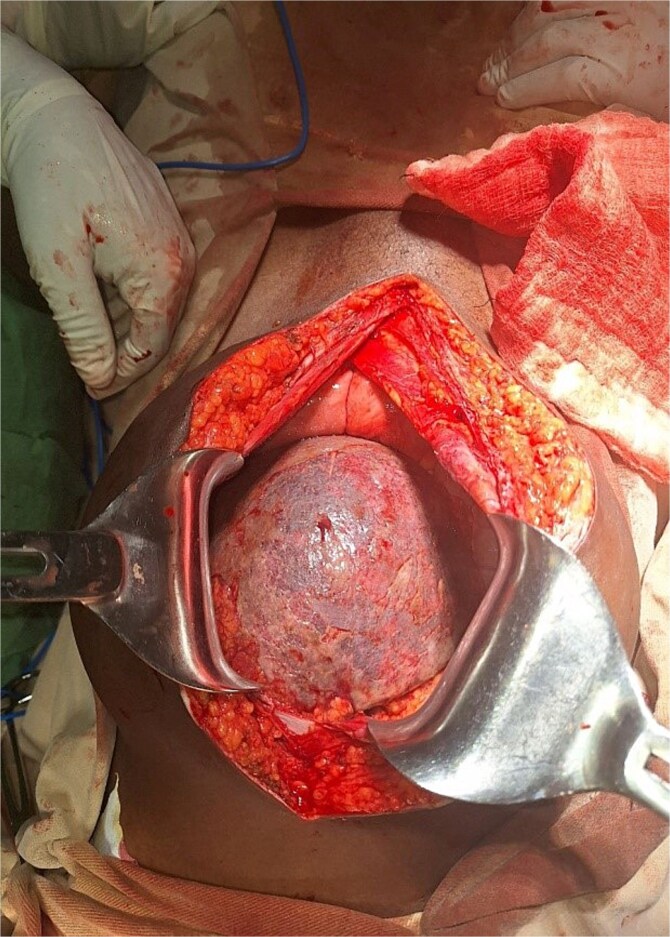
Pelvic spleen with visibly ischemia up on entering to abdomen.

**Figure 2:**
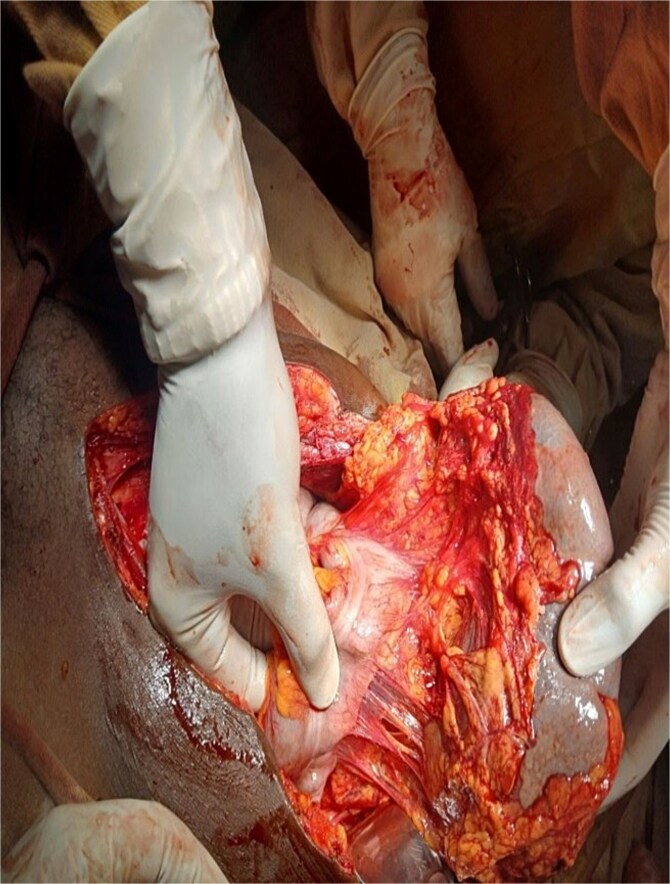
Detorsed spleen with pedicle arising from left gasteroepiploic vessels.

An exploratory laparotomy was performed for suspected adnexal mass torsion. Intraoperatively, an enlarged, ischemic pelvic spleen was found, torsed 540° counterclockwise, with no attachments to the phrenicosplenic or splenorenal ligaments (Figs 1 and 2). The spleen was ectopic, located in the greater omentum, with its hilar blood supply arising from the left gastroepiploic artery. Approximately 100 ml of reactive fluid was also present.

**Figure 3:**
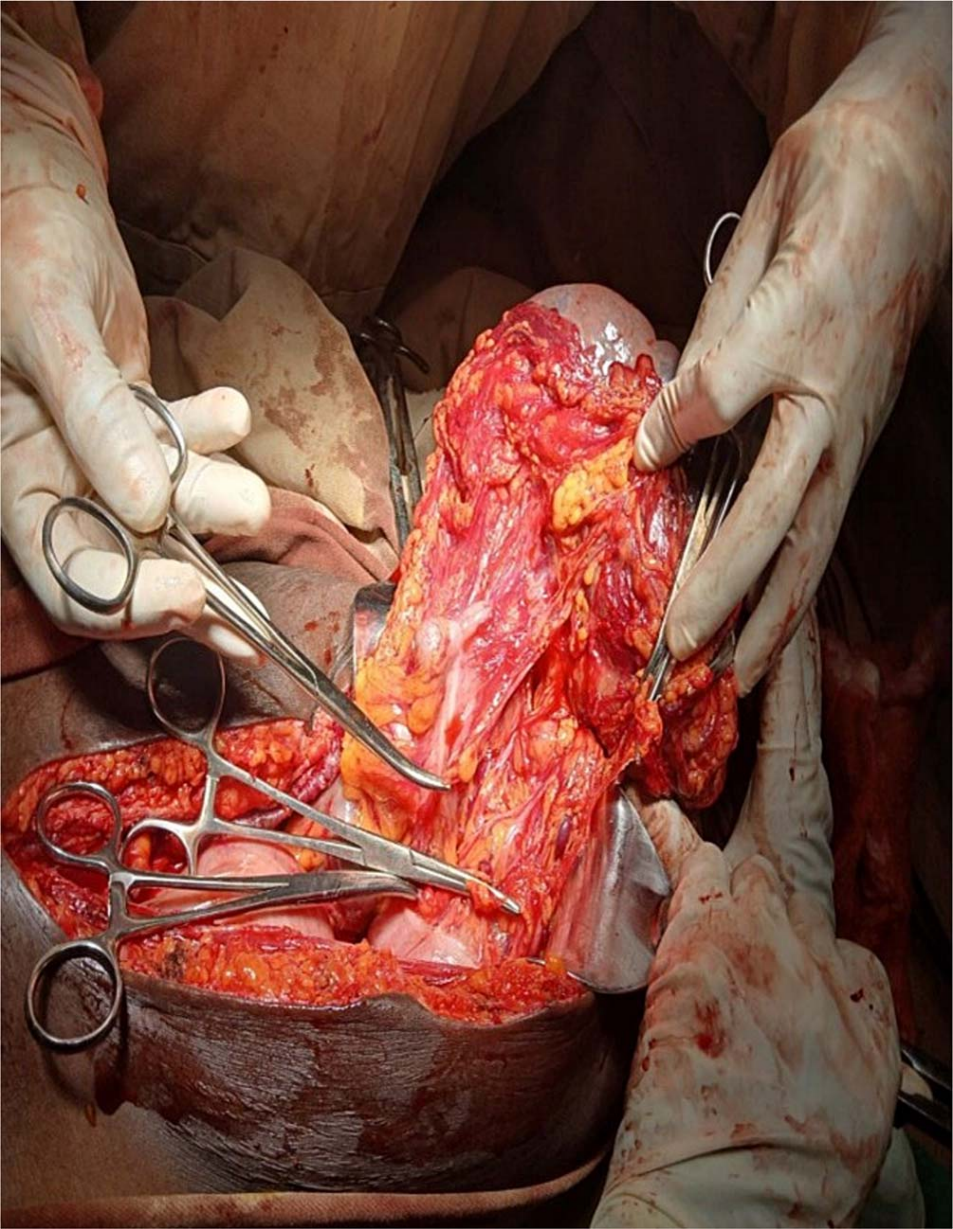
Left gasteroepiploic vessels being ligated.

**Figure 4:**
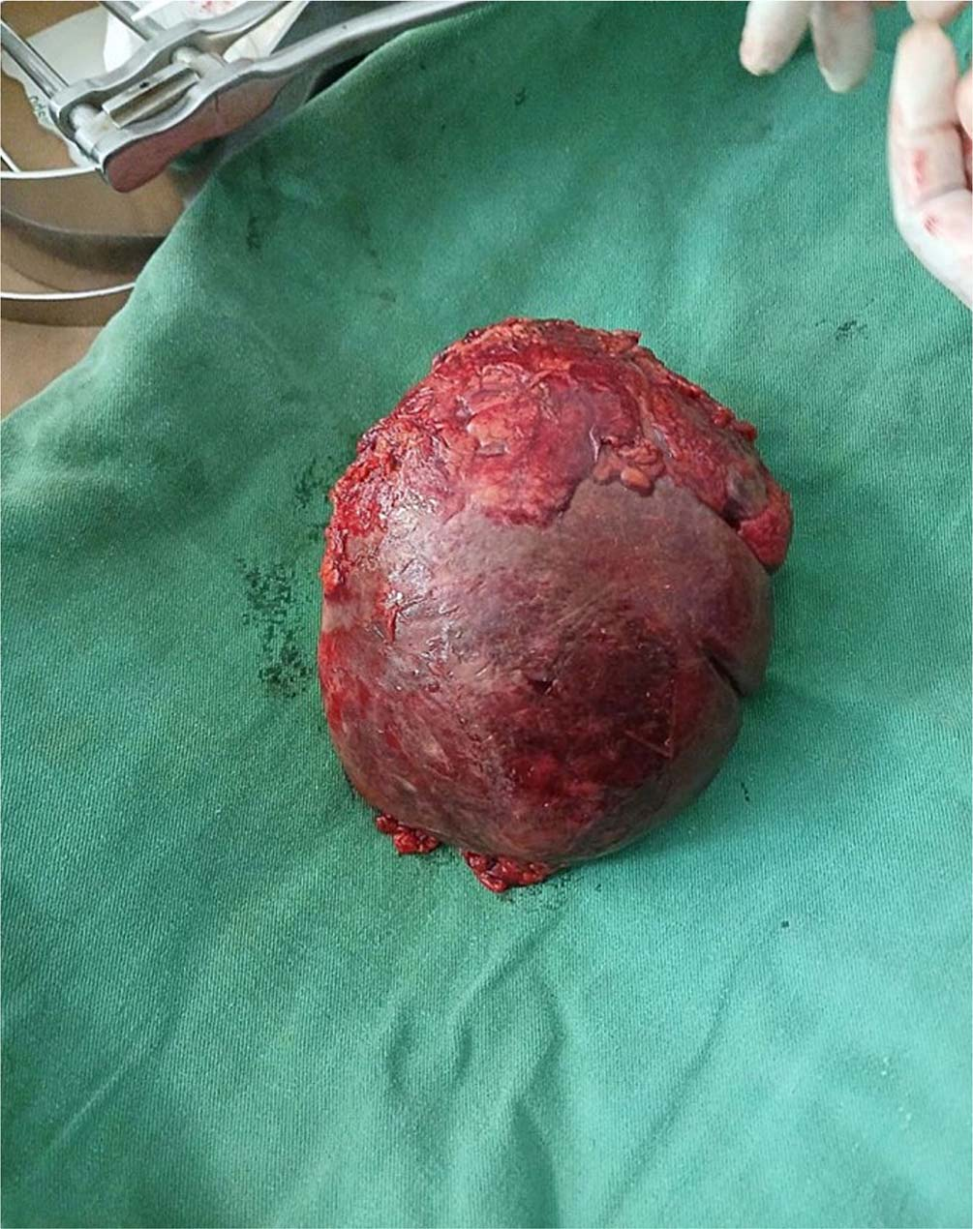
The spleen after removal.

After detorsion and identification of the splenic vessels and ligaments, an open splenectomy was performed (Figs 3 and 4). She had an uneventful postoperative recovery and was discharged on the fourth day. At her 2-week follow-up, she remained asymptomatic and received post-splenectomy vaccinations.

## Discussion

Wandering spleen is a rare medical entity in which a normal spleen is hypermobile and ectopic either from congenital or acquired causes [1, 2]. Congenitally, it is due to anomalies in the development of the dorsal mesogastrium and splenic suspensory ligaments particularly the gastrosplenic and splenorenal ligaments [3]. Acquisitively, it can result from hormonal effects of pregnancy and abdominal wall laxity, making it common to be encountered in the reproductive women aged 20–40 years though it mainly affects children in about third of cases from the general population [4, 5]. Other acquired causes include splenomegaly, trauma and diaphragmatic hernia repair [4]. All etiologies result in a long pedicle which is at risk for torsion [3, 6]. It is estimated to be the cause for splenectomies in ⁓0.2% of cases [7].

In most cases, patients are asymptomatic. But, they can also present either with chronic symptoms or emergently as in our case. The diagnosis can be suspected when there is a firm, mobile, notched abdominal mass elsewhere than the left hypochondrium. However, the notch can be hidden with engorgement [2]. With complications, patients usually present with acute abdominal often in the lower quadrants. They can have diffuse tenderness as in our case. Torsion is the most common complication, which leads to impaired arterial supply preceded by venous stasis and congestion resulting in splenic infarction and necrosis [3, 7].

Abdominal radiograph has limited role. It may show displaced bowel loops by a soft tissue density [8]. In a normally positioned spleen, ultrasonography (US) shows a homogeneous mass in the left upper quadrant, slightly hyperechoic relative to the renal cortex and slightly hypoechoic or isoechoic compared to the liver parenchyma [2]. But, in splenic torsion, it shows absence of spleen in its normal anatomic position with a comma shaped structure located elsewhere in the abdomen or pelvis [2]. It became heterogenous when get infarcted and doppler can reveal decreased or absent flow with splenic vein thrombosis. But, if the torsion is incomplete or partially necrotic, it might have flow [1]. In our case, we actually missed to find the absence of left hypochondrium spleen, but the US detected a hypoechogenic mass in the pelvic region with cystic component, which might suggest the infarction we got.

Multislice spiral CT of the abdomen is helpful to detect an earlier stage splenic torsion before infarction which is important to preserve it [9]. CT scan should be considered when the US is negative or equivocal [1]. It can reveal abnormally vertically aligned spleen with ectopic position [1]. The ‘whirl’ sign is pathognomonic [8]. Infarcted spleen can be partially or non-enhancing with a white rim (“rim sign”) [2]. In our setting, CT scan was temporarily unavailable which make us to just rely on our physical finding and US to explore.

Surgical exploration is warranted with suspected or diagnosed splenic torsion [6]. If there is no previous abdominal surgery laparoscopy is the preferred method [10]. If the spleen is not infarcted splenopexy should be the option, particularly in the pediatrics [11]. There are different methods of splenopexy mentioned in the literature [12]. We did splenectomy since the spleen was frankly necrotic.

## Conclusion

Torsion of a wandering spleen is a rare and often misdiagnosed condition, particularly in female patients presenting with acute abdominal pain. Imaging, particularly CT scans, is crucial for accurate diagnosis, though ultrasound remains a valuable tool in resource-limited settings. Early surgical exploration is essential to salvage the spleen or prevent complications.
